# Succinylcholine relaxant: Anaesthesiologist not relaxed!

**DOI:** 10.4103/0019-5049.60508

**Published:** 2010

**Authors:** Sarah Ninan, L Jeslin, PA Saravanan, Kamal Kumar

**Affiliations:** Department of Anesthesia and Critical Care, Christian Medical College and Hospital, Vellore-632 004, India

Sir,

Succinyl choline (Sch) remains the relaxant of choice for emergency surgery. However, in some patients, prolonged apnoea can occur.[1] We report a child who had an unanticipated prolonged apnoea following Sch administration and the role of intraoperative fresh frozen plasma administration.

A nine-year-old boy (weight: 23 kg) ASA I, was scheduled for emergency exploration in view of torsion of testis. His medical history, clinical examination and baseline blood investigations were essentially normal. Patient had last meal four hours prior to surgery. In the operating room, a standard rapid sequence induction technique was performed using thiopentone 5 mg/kg and succinylcholine 1 mg/kg under standard monitoring. Trachea was intubated and ventilated. Injection Morphine 1 mg, fentanyl 50 mcg, paracetamol 400 mg were used as analgesics. Intraoperative vital signs were stable. Anaesthesia was maintained with oxygen, air and 1% isoflurane. Non-depolarizing muscle relaxant was not administered and the duration of surgery was 45 minutes. There were no clinical signs of spontaneous breathing even after 30 minutes. Train of four and post tetanic count stimuli using nerve stimulator revealed no response.

A provisional diagnosis of scoline apnoea was made after ruling out other possible aetiologies. Serum pseudo cholinesterase level estimation was sent immediately and it was 71 U/L, well below the normal value (3000 6000 U/L). Senior anaesthesiologist and haematologist were consulted and decided to transfuse fresh frozen plasma (15 ml/kg) and to proceed accordingly. About five minutes after transfusion (spontaneous breathing returned. Patient was extubated awake, with a positive five-second head lift test. Postoperatively, he was monitored in high dependency unit and discharged with warning card after two days.

The duration of neuromuscular blockade after normal doses of Sch (1.0-1.5 mg/kg) is usually four to six minutes.[2] Delayed or prolonged metabolism of Sch may cause prolonged apnoea. It is usually due to abnormal butrylcholinesterase (BChE), severe hepatic dysfunction, interactions with other drugs that inhibit BChE.[3] The cholinesterase gene is located on chromosome 3 at q26, 40 and 20 mutations in the coding region of the cholinesterase gene have been identified. Patients who are homozygous (approximately 1:2,000 individuals) have prolonged paralysis (three to six hours) and who are heterozygous (1:30 cases), the duration of action is only slightly prolonged.[4] Pantuck and Pantuck recommended mild hyperventilation and light anaesthesia maintenance until spontaneous recovery.[1] Viby-Morgenson suggested cholinesterase can be administered either in the form of fresh-frozen plasma or a cholinesterase concentrate 90-130 mg.[5] Patient was breathing adequately, maintained vitals following fresh frozen plasma administration. Serum cholinesterase was not measured as he was clinically stable.

Thus prolonged apnoea following Sch administration needs early recognition, intraoperative administration of fresh frozen plasma when available and monitoring in high dependency unit in the postoperative period. When FFP or cholinesterase concentrates are not available, elective ventilation is the only option, as described in literature.

## References

[1] Pantuck EJ, Pantuck CB, Azar I (1987). Prolonged apnea following succinylcholine administration. Muscle relaxants.

[2] Meakin G, McKiernan EP, Morris P, Baker RD (1989). Dose-response curves for suxamethonium in neonates, infants and children. Br J Anaesth.

[3] Calvey TN, Williams NE, Calvey N, Williams N (2008). Drugs that act on the neuromuscular junction. Principles and practice of pharmacology for anaesthetists.

[4] Rosenberg H, Brandom BW, Barash PG, Cullen BF, Stoelting RK (2006). Malignant hyperthermia and other pharmacogenetic disorders. Clinical anesthesia.

[5] Viby-Morgenson J (1981). Succinylcholine neuromuscular blockade in subjects homozygous for atypical plasma cholinesterase. Anesthesiology.

